# Periprocedural outcome in patients undergoing left atrial appendage occlusion with the Watchman FLX device: The ITALIAN-FLX registry

**DOI:** 10.3389/fcvm.2023.1115811

**Published:** 2023-04-27

**Authors:** Sergio Berti, Alberto Ranieri De Caterina, Carmelo Grasso, Gavino Casu, Giuseppe Giacchi, Paolo Pagnotta, Michele Maremmani, Patrizio Mazzone, Luca Limite, Francesco Tomassini, Francesco Greco, Maria Rita Romeo, Giuseppe Caramanno, Gaetano Fassini, Salvatore Geraci, Mauro Chiarito, Claudio Tondo, Corrado Tamburino, Marco Contarini

**Affiliations:** ^1^ Fondazione Toscana G. Monasterio, Ospedale del Cuore G. Pasquinucci, Massa, Italy; ^2^ Cardiac-Thoracic-Vascular Department, Ferrarotto Hospital and University of Catania, Catania, Italy; ^3^ Cardiology Unit, Ospedale San Francesco, Nuoro, Italy; ^4^ Cardiology Department, Umberto I Hospital, Syracuse, Italy; ^5^ Department of Biomedical Sciences, Humanitas University, Pieve Emanuele, Milan, Italy; ^6^ Cardio Center, Humanitas Research Hospital IRCCS, Rozzano, Milan, Italy; ^7^ Department of Cardiac Electrophysiology and Arrhythmology, IRCCS San Raffaele Hospital and Vita-Salute University, Milan, Italy; ^8^ Unità interaziendale di emodinamica-Ospedale degli Infermi, Rivoli(To)-Ospedale San Luigi Gonzaga, Turin, Italy; ^9^ Cardiology Department, A.O. “Annunziata”, Cosenza, Italy; ^10^ Department of Clinical Electrophysiology & Cardiac Pacing Heart Rhythm Center at Monzino Cardiac Center, IRCCS, Milan, Italy; ^11^ Interventional Cardiology Unit, Umberto I Hospital, Syracuse, Italy

**Keywords:** left atrial appendage occlusion, atrial fibrillation, Watchman FLX, ischemic stroke, stroke prevention

## Abstract

**Introduction:**

The Watchman FLX is a novel device for transcatheter left atrial appendage occlusion (LAAO) specifically designed to improve procedural performance in more complex anatomies with a better safety profile. Recently, small prospective non-randomized studies have shown good procedural success and safety compared with previous experiences. Results from large multicenter registries are needed to confirm the safety and efficacy of the Watchman FLX device in a real-world setting.

**Methods:**

Italian FLX registry is a retrospective, non-randomized, multicentric study across 25 investigational centers in Italy including consecutive patients undergoing LAAO with the Watchman FLX between March 2019 and September 2021 (N = 772). The primary efficacy outcome was the technical success of the LAAO procedure (peri-device flow ≤ 5 mm) as assessed by intra-procedural imaging. The peri-procedural safety outcome was defined as the occurrence of one of the following events within 7 days after the procedure or by hospital discharge: death, stroke, transient ischemic attack, major extracranial bleeding (BARC type 3 or 5), pericardial effusion with tamponade or device embolization.

**Results:**

A total of 772 patients were enrolled. The mean age was 76 ± 8 with a mean CHA2DS2-VASc score of 4.1 ± 1.4 and a mean HAS-BLED score of 3.7 ± 1.1. Technical success was achieved in 772 (100%) patients with the first device implanted in 760 (98.4%) patients. A peri-procedural safety outcome event occurred in 21 patients (2.7%) with major extracranial bleeding being the most common (1.7%). No device embolization occurred. At discharge 459 patients (59.4%) were treated with dual antiplatelet therapy (DAPT).

**Conclusions:**

The Italian FLX registry represents the largest multicenter retrospective real-world study reporting periprocedural outcome of LAAO with the Watchman FLX device, resulting in a procedural success rate of 100% and a low incidence of peri-procedural major adverse events (2.7%).

## Background

Transcatheter left atrial appendage occlusion (LAAO) represents a valuable therapeutic option for stroke prevention in patients with non-valvular atrial fibrillation (NVAF) (1, 2). Among the available percutaneous left atrial appendage (LAA) closure device, Watchman™ (Boston Scientific Inc) is the only device for which randomized trials have shown comparable efficacy and safety with a long-term vitamin-k antagonist (VKA) for the prevention of stroke and systemic embolization (3–5).

First-generation devices for LAAO, including Watchman, provide a high technical success rate and relatively low procedure-related adverse events incidence (6, 7). However, there are still some limitations, especially in more complex LAA anatomies. The novel Watchman FLX device has been designed to overcome these limitations allowing simplified implantation on a wider range of LAA anatomies. Some observational studies reported a low incidence of adverse events and a high incidence of anatomic closure with the new-generation device (8–12). However, only a few numbers of patients were enrolled and data reflecting real-world experience are missing. More importantly, the exclusion of patients with contraindication to oral anticoagulation (8) limits the possibility to generalize these results to those patients who would benefit most from LAAO.

This study aims to report the largest experience of LAAO using the novel generation Watchman FLX device in an all-comers consecutive cohort of patients with AF at high bleeding risk.

## Methods

The ITALIAN FLX Registry is a retrospective, non-randomized, multicentric study across 25 investigational centers in Italy enrolling all consecutive patients undergoing LAAO with the Watchman FLX between March 2019 and September 2021 (*N* = 772). The registry intended to monitor the early and late performance of the Watchman FLX devices regardless of the specific protocol for implantation used in each institution. Therefore, there were no restrictions to any given personal or institutional protocols with respect to the indications, pre-procedural planning, device implantation, drug therapy, and follow-up. Informed consent was obtained from each patient, and the study protocol conforms to the ethical guidelines of the 1975 Declaration of Helsinki as reflected in *a priori* approval by the institution's human research committee.

### WATCHMAN FLX device

The new-generation WATCHMAN FLX device for transcatheter LAAO was designed to simplify implantation on a broader range of LAA anatomies (13). Compared to the previous generation of WATCHMAN devices, it is characterized by (1) less device height allowing improved handling in shallow left atrial appendage (LAA) anatomies; (2) a closed distal device end aiming for less traumatic and capable of full recapture and repositioning; (3) a recessed screw with reduced metal surface area, possibly reducing DAT; (4) an increased number of hooks, from 10 to 18, redistributed into two rows with a J-shaped form to minimize trauma to the appendage wall, possibly reducing pericardial effusion.

### Study protocol

Clinical characteristics and clinical indications to LAAO were reported for each patient. Specifically, CHA_2_DS_2_-VASc score (congestive heart failure, hypertension, age ≥75 years, diabetes mellitus, stroke/transient ischemic attack, vascular disease, age 65–74 years, sex category) as well as HAS-BLED score (hypertension, abnormal renal/liver function [1 or 2 points], stroke, bleeding history or predisposition, labile international normalized ratio, elderly [>65 years], drugs/alcohol concomitantly) were accurately collected. All patients underwent pre-procedural imaging with cardiac computed tomography (CT) or transesophageal imaging (TEE) as per standard institutional practice to exclude LAA thrombosis and obtain accurate device sizing. The pre-procedural acquisition protocol has been described previously in detail (14–16). No anatomical variant was excluded from the closure attempt.

The primary efficacy outcome was the technical success of the LAAO procedure,defined as successful deployment and implantation of the device with any peri-device flow with jet size ≤5 mm as assessed by intra-procedural imaging. The periprocedural safety outcome was defined as the occurrence of one of the following events within 7 days after the procedure or by hospital discharge: death, stroke, transient ischemic attack, major extracranial bleeding (Bleeding Academic Research Consortium type 3 or higher), pericardial effusion with tamponade or device embolization. All outcome variables were defined according to the Munich consensus document on definitions and endpoints in LAAO (17). The optimal antithrombotic treatment at discharge was individualized on patient-specific characteristics balancing the thrombotic and hemorrhagic risk; when safe, patients were discharged on dual antiplatelet therapy (DAPT) regimen for up to 6 months.

Follow-up reflected schedules of each enrollment site. Generally, an initial visit was planned at 1–3 months, followed by a routine follow-up at 1 year. All data collection and adverse event reporting was performed directly by the individual sites and captured in a standardized central database. Clinical outcomes during follow-up were reported as total events and incidence rate of all-causedeath, stroke, and major bleeding. Adverse events such as death, stroke, major bleeding, cardiac tamponade, and device thrombosis were monitored during follow-up. Bleeding was assessed according to the BARC (Bleeding Academic Research Consortium) criteria (18). Stroke was classified according to the criteria defined by Leon et al. (19). All data and events were adjudicated and entered into the central database by the local investigators. Verification of source data was carried out during site visits, and for major adverse events, the source documents were requested at the sites and reviewed remotely. These included as a minimum death from all causes, all strokes, TIA, systemic embolism (SE), and all adverse events occurring within 7 days of implantation, regardless of whether the event was judged to be severe and whether or not it was related to the device/procedure.

### Statistical analysis

Descriptive statistics were used, with continuous variables expressed as mean ± SD or median [interquartile range (IQR)] as appropriate. Categorical variables are expressed as absolute numbers and proportions.Significance between the groups was assessed by analysis of the student *t*-test. In case two or more variables were compared, Bonferroni-adjusted significance was used.

## Results

### Study population

Clinical characteristics of the population are reported in Table 1. A total of 772 were enrolled during the study period. The mean age was 76 ± 8 with almost two-thirds of patients being male (66%). Of note, more than one-third of patients (36.1%) had a history of previous cerebrovascular accidents. The mean CHA_2_DS_2_-VASc was 4.1 ± 1.5 and the mean HAS-BLED score of 3.7 ± 1.1. A history of major bleeding was present in 440 patients (57%) and one out of six patients underwent LAAO due to severe chronic kidney disease contraindicating oral anticoagulation.

**Table 1:** Baseline characteristics (*N* 772).

Characteristics	All enrolled (*n* = 772)
Age (years)	76.5 ± 8.3
Age ≥80 years old, *n* (%)	308 (39.8)
Male gender, *n* (%)	507 (66)
Body Mass Index, Kg/m^2^	26.6 ± 4
Arterial hypertension, *n* (%)	576 (74.6)
Diabetes, *n* (%)	244 (31.6)
Ischemic heart disease, *n* (%)	292 (37.8)
Prior ischemic stroke, *n* (%)	99 (12.8)
Prior Hemorrhagic stroke, *n* (%)	123 (15.9)
Prior TIA, *n* (%)	65 (8.4)
Cancer	63 (8.2)
Glomerular filtration rate, ml/min	56 (39–75)
LVEF, %	55 (45–60)
Permanent Atrial Fibrillation	371 (48)
CHA2DS2-VASc score	4.1 ± 1.5
CHA2DS2-VASc score ≥4	518 (67.1)
HAS-BLED score	3.7 ± 1.1
HAS-BLED score ≥3	667 (86.4)
OAC contraindication	533 (69.0%)
Primary indication for LAA occlusion	
Gastrointestinal bleeding	234 (30.3)
Intracranial bleeding	110 (14.2)
Urogenital bleeding	15 (1.9)
Other Spontaneous bleeding	85 (11)
Other indication	
Severe CKD or liver disease	121 (15.7)
Hematologic disorder	69 (8.9)
Ischemic stroke despite OAC	51 (6.6)
Need for triple antithrombotic therapy or prolonged DAPT after PCI	7 (0.9%)
Cerebrovascular malformation	6 (0.8)
Cerebral Amyloid Angiopathy	9 (1.1)

Values are mean ± SD, *n* (%), or median (interquartile range).

CKD, chronic kidney disease; DAPT, dual anti-platelet therapy; LAA, left atrial appendage; LVEF, left ventricular ejection fraction; N, number; OAC, oral anti-coagulation; PCI, percutaneous coronary intervention; TIA, transient ischemic attack.

### Procedural success

Procedural characteristics are presented in Table 2. Technical success was achieved by 772 (100%) patients with the first selected device implanted in 760 (98.4%) patients. No intraprocedural device embolization occurred. Device implantation and procedure were mainly driven by fluoroscopy and TEE (83.2%) or by intracardiac echocardiography (18.9%); the comprehensive median procedure time was 60 min (38–80 min). The median hospital length of stay was 4 (3–5) days.

**Table 2:** Procedural characteristics (*N* 772).

Size of implanted device	
FLX 20	99 (12.8)
FLX 24	201 (26.0)
FLX 27	211 (27.3)
FLX 31	153 (19.8)
FLX 35	91 (11.8)
First device implanted	760 (98.4)
Procedure time, min	60 (38–80)
Contrast use, ml	80 (55–110)
Fluoroscopy time, min	15 (10–23)
Procedural guidance	
Intracardiac echocardiography	146 (18.9%)
Transesophageal echocardiography	642 (83.2)
Length Of Admission, Days	4 (3–5)

Values are mean ± SD, *n* (%), or median (interquartile range).

FLX, Watchman Flex device; N, number.

### Periprocedural safety

Peri-procedural adverse events are summarized in Table 3. A periprocedural safety outcome event occurred in 21 patients (2.7%), with major extracranial bleeding being the most common (1.7%). Among the major extracranial bleedings (BARC type 3 or 5), 6 were related to femoral venous access (0.77%), with only 1 patient requiring vascular surgery due to the presence of a pseudoaneurysm. We also observed 1 case of genito-urinary bleeding due to a Foley catheter insertion, 1 abdominal hemorrhage due to the presence of a uterine polyp, 1 epistasis, and 1 case of massive gastrointestinal bleeding leading to death; the latter was an 83-year-old man with multiple comorbidities (hypertension and diabetes) and a history of gastrointestinal bleeding, impaired renal function, ischemic heart disease (previous MI and multiple PCI) and an elevated thrombotic and hemorrhagic profile risk (CHA_2_DS_2_-VASc score of 5—HAS-BLED score of 6) who experienced a massive post-procedural gastro-intestinal bleeding with hemorrhagic shock which led to death 2 days after the index procedure.Two patients (0.3%) experienced a peri-procedural pericardial tamponade requiring percutaneous drainage.

**Table 3:** Peri-procedural complications (*N* 772).

Type of complication	*N* (%)
Death	3 (0.4)
Ischemic stroke	1 (0.1)
Intracranial hemorrhage	1 (0.1)
TIA	1 (0.1)
Major extracranial bleeding (BARC type 3 or 5)^a^	13 (1.7)
Pericardial effusion with tamponade	2 (0.3)
Device embolization	0 (0.0)
Device-related complications	1 (0.1)
Procedure-related complications	10 (1.3)

Values are *n* (%).

BARC, bleeding academic research consortium; ICH, intra-cranial hemorrhage; n, number; TIA, transient ischemic attack.

^a^ Not including pericardial tamponade and ICH.

We also observed 2 cases of periprocedural stroke (0.3%). One patient experienced a periprocedural hemorrhagic stroke. The patient was a 79 years old diabetic man with a history of intracranial hemorrhage, impaired renal function, and carotid disease. During the procedure, he experienced a subarachnoid hemorrhage without surgical indication. After clinical stabilization, he was discharged after 7 days from the index procedure without antithrombotic treatment due to the prohibitive bleeding risk. One patient (0.1%) presented with aphasia and right sensitivity and motor deficit two days after the procedure due to a left hemisphere ischemic stroke confirmed by the computed tomography scan. The TEE showed a device-related thrombosis (DRT) despite early initiation of DAPT; thus, after a 48 h safety window, the patient switched to aspirin plus VKA. After 30 days of rehabilitation, the patient was discharged with full neurological recovery and any DRT revealed by TEE.

Peri-procedural death was observed in 3 patients. One patient, an 80-year-old diabetic female with impaired renal function and chronic heart failure with ascitis, experienced an arrhythmic death 3 days after the index procedure. However, echocardiography did not reveal LAAO device migration or DRT, so the link between LAAO procedure and death appears elusive. A second patient, previously described, died as a consequence of a massive post-procedural gastro-intestinal bleeding with hemorrhagic shock 2 days after the index procedure. A third patient, an 85-year-old man with advanced chronic heart failure, died 14 days after the index procedure due to refractory cardiogenic shock.

Collectively, we categorized 10 (1.3%) procedure-related complications (6 cases of access-related bleeding, 2 cases of pericardial hemorrhage and 2 cases of periprocedural stroke), with only 1 (0.1%) classified as a device-related complication (1 DRT).

### PostLAAO antithrombotic therapy

At discharge, a total of 459 patients (59.4%) were treated with DAPT (aspirin plus clopidogrel), 111 (14,4%) with a combination of low molecular weight heparin (LMWH) plus SAPT, 96 (12.4%) with SAPT, 34 (4.4%) with LMWH alone, 24 (3.1%) with NOAC alone, 4 (0.3%) with VKA alone and 44 (5.6%) patients with any antithrombotic therapy due to prohibitive risk of bleeding. The mean time indication for dual antithrombotic treatment at discharge was 3.4 ± 3.3 months.

## Discussion

The present study represents the largest multicenter prospective study to report the efficacy and safety outcomes of the novel WATCHMAN Flex device for transcatheter LAAO. Our major findings are that the procedure of LAAO using the WATCHMAN Flex device was associated with a procedural success rate of 100% irrespective of LAA anatomy. The observed incidence of periprocedural safety events of 2.7%. The incidence of peri-procedural major bleeding (BARC type 3 or 5) was 1.8% with major extra-cranial bleeding being the most common (*N* = 13; 1.7%). Overall, 2 patients experienced pericardial effusion requiring percutaneous drainage (0.3%).

### A real high-risk population

Although still confined into a recommendation of class IIb both in European and American Guidelines (1, 20) LAAO represents a valuable option to reduce cardioembolic risk in patients with AF and high bleeding risk and/or contraindication to oral anticoagulation. A recent meta-analysis including three randomized open-label controlled trials (Protect-AF, Prevail, and Prague-17), demonstrated that LAAO with the Watchman device had a similar rate of stroke when compared with those for OAC (VKA or NOAC) in the prevention of thromboembolism in NVAF, with observed reduction in hemorrhagic stroke, cardiovascular and all-cause death (5). The population enrolled in the Italian Flex registry represents a particularly high-risk subset of patients, at higher bleeding risk compared to what is currently reported in the literature but in line with patients routinely referred to LAAO in our everyday practice. In fact, while CHA2DS2-VASc score was similar compared to other registries or metanalysis, including those using the Watchman FLX device (8–12) in our population, we observed a mean HAS-BLED score of 3.7—much higher than that reported, for instance, in the EWOLUTION trial with Watchman device (2.1) (7), in the Amulet Observational registry by Landmesser (3.3) (21) and in previous experience with Watchman FLX device (2.4) (9). Of the total of 772 patients, 518 (67.1%) had a CHA2DS2-VASc score ≥4 and 667 (86.4%) had a HAS-BLED score ≥3. Moreover, 553 patients (69%) had an absolute or relative contraindication to oral anticoagulation therapy.

### Procedural success

In our study procedural success was achieved in 100% of cases. Of note, the implantation was performed with the first selected device implanted in 98.4% of cases. This very high rate of successful implantation represents the highest ever reported in an all-comers high-risk population undergoing LAAO. In the initial experience with the Watchman device, technical success was achieved in 88% in the PROTECT-AF trial, then it progressively increased over time to 95.1% in the PREVAIL trial and 98.5% in the EWOLUTION trial. Similarly, our reported technical success appears to be higher than that reported with the Amulet device (97.3% as found by Tsikas etal and 99% in the Prospective Global AMPLATZER Amulet Observational Study). In the PINNACLE study (8) as well in the initial European experience (9–11)—reporting the initial experience with the Watchman FLX device—technical success approximated 100% of cases (100% in 3 cases and 99% in the fourth), but collectively these 4 studies did not reach the numerosity of the Italian FLX registry (*n* = 400, 165, 38 and 91, respectively vs. *n* = 772 of the Italian FLX registry).

The progressive increase of technical success over time in the field of LAAO is the result of the learning curve of the operators combined with the increased reliance on pre-procedural imaging and planning.On the other hand, the technological refinement of devices plays a major role. In this regard, the novel Watchman Flex has been designed to provide an improved conformability, a full or partial recapture during implant, better device stability and maneuverability within the appendage as well as a shorter length and wider sizing matrix to treat a wider variety of anatomies. These technical characteristics have clearly influenced the technical success first observed in the PINNACLE (8) and in the studies from Korsholm et al. (9), Grygier et al. (10) and Cruz-González et al. (11) and now confirmed by our data. In addition, no intraprocedural device embolization occurred.The innovative design with two rows of anchors and the possibility to fully recapture and reposition the device allowing for optimal deployment are both aspects that may have contributed to this remarkable outcome.

### Periprocedural safety

The reported incidence of a periprocedural safety event using the WATCHMAN FLX, ranges from 0.5% to 5.5% (8, 11); such variability may be partly related to differences in the baseline risk of bleeding as well as the variable sample size. The PINNACLE FLX trial, the largest prospective study available at this moment (400 patients), reported an incidence of a periprocedural safety event of 0.5% which is lower than that observed in ITALIAN FLX (2.7%) [8]. However, it should be noted that patients enrolled in this trial had a relatively low risk of bleeding (mean HAS-BLED score of 2.0 ± 1.0) and a history of major bleeding in only 33% of participants.Moreover, patients enrolled in the PINNACLE were prescribed a DOAC for at least 45 days [8]. Conversely, patients enrolled in the Italian FLX were representative of a particularly high bleeding risk population having a higher mean HAS-BLED score (3.7 ± 1.1, *p* < 0,0001)and a higher rate of previous major bleeding (PINNACLE FLX 33% vs. ITALIAN FLX 46.4%, *p* < 0.0001). Of note, we observed 3 cases of periprocedural death. Only one of these cases might be theoretically related to the procedure—an arrhythmic death 3 days after the procedure. In the other 2 cases—one hemorrhagic shock after gastro-instestinal bleeding and one cardiogenic shock 2 weeks after LAAO- the final cause of death seems to be related more to the extreme patient's frailty rather than to the procedure itself.

Our observed procedural safety is in line with that observed in the EWOLUTION study, the largest prospective multicenter registryreflecting real-world experience with the previous generation WATCHMAN device (7). Such study enrolled 1021 patients undergoing LAAO, with a mean age of 73.4 ± 8.9 years, a mean HAS-BLED score of 2.3 ± 1.2. Oral anticoagulation was contraindi­cated in 62% of patients and 45% had suffered a prior stroke. A periprocedural major adverse cardiac event was seen in 18 patients (1.8%); in particular: 9 major bleeding (BARC type 3 or 5),3 cardiac tamponades,4 deaths, and 2 device embolization. These results, representing real-life clinical outcomes with the predecessor WATCHMAN device, were consistent with our findings (EWOLUTION 1.8% vs. ITALIAN FLX 2.7%, *p* > 0,05).

Periprocedural stroke occurred in 2 patients (0.2%), one was ischemic (0.1%) and 1 was hemorrhagic (0.1%). The hemorrhagic stroke appeared unrelated to the device, but as a result of the high hemorrhagic risk of the patient who presented with a positive history of major intracranial bleeding. Conversely, the ischemic stroke was procedure-related, as TEE showed a DRT two days after the procedure. The patient was treated with dual antiplatelet therapy soon after the procedure. The EWOLUTION study reported an incidence rate of DRT of 4.1%, but recent data have downgraded its incidence to 1.7%/year (22). The postimplant antithrombotic strategy observed in our registry was highly variable and representative of a real-world scenario. DAPT was chosen in 60% of patients. The post-procedural anti-thrombotic treatment strategy observed in our registry was in line with this finding as 459 patients (59.4%) were treated with DAPT at discharge (EWOLUTION 60% vs. ITALIAN FLX 59%, *p* > 0.05).

#### Study limitation

The current registry has several weaknesses. First, retrospective design has intrinsic limitations, lacking a randomized group and leaving it vulnerable to bias and misunderstanding. Second, caution should be made when comparing outcomes between populations with different baseline characteristics, especially in the absence of a propensity-matched score analysis. Third, the multicentric design of the registry limits the possibility of a standardized post-procedural anti-thrombotic management as well as scheduled follow-up and data collection. Although high- and low-volume centers coexist in the current registry, we didn't observe significant differences between the two in terms of procedural success, nor peri-procedural adverse events. Nevertheless, the study cohort represented a real-world population of NVAF patients at highrisk of stroke and bleeding encountered in daily practice. Follow-up data, soon available, will be essential to confirm the long-term safety and effectiveness of the device, as well as the incidence rate of DRT.

## Conclusion

In this large, multicenter retrospective real-world cohort, LAA occlusion with the Watchman FLX device was associated with a procedural success rate of 100% and a low incidence of peri-procedural major adverse events (2.7%).

## Data Availability

The raw data supporting the conclusions of this article will be made available by the authors, without undue reservation.
